# Supplementary material for: High-performance chemical- and light-inducible recombinases in mammalian cells and mice
Source: Nat Commun. 2019 Oct 24;10:4845. doi: 10.1038/s41467-019-12800-7 (PMC6813296; doi:10.1038/s41467-019-12800-7)
Supplement: Supplementary file 3 — Reporting Summary [file 41467_2019_12800_MOESM3_ESM.pdf]

nature research

Corresponding author(s): Wilson Wong

# Reporting Summary

Nature Research wishes to improve the reproducibility of the work that we publish. This form provides structure for consistency and transparency in reporting. For further information on Nature Research policies, see Authors & Referees and the Editorial Policy Checklist.

## Statistical parameters

When statistical analyses are reported, confirm that the following items are present in the relevant location (e.g. figure legend, table legend, main text, or Methods section).

| n/a | Confirmed | |
|---|---|---|
| | x | The exact sample size (*n*) for each experimental group/condition, given as a discrete number and unit of measurement |
| | x | An indication of whether measurements were taken from distinct samples or whether the same sample was measured repeatedly |
| x | | The statistical test(s) used AND whether they are one- or two-sided<br>*Only common tests should be described solely by name; describe more complex techniques in the Methods section.* |
| x | | A description of all covariates tested |
| x | | A description of any assumptions or corrections, such as tests of normality and adjustment for multiple comparisons |
| | x | A full description of the statistics including central tendency (e.g. means) or other basic estimates (e.g. regression coefficient) AND variation (e.g. standard deviation) or associated estimates of uncertainty (e.g. confidence intervals) |
| x | | For null hypothesis testing, the test statistic (e.g. *F*, *t*, *r*) with confidence intervals, effect sizes, degrees of freedom and *P* value noted<br>*Give P values as exact values whenever suitable.* |
| x | | For Bayesian analysis, information on the choice of priors and Markov chain Monte Carlo settings |
| x | | For hierarchical and complex designs, identification of the appropriate level for tests and full reporting of outcomes |
| x | | Estimates of effect sizes (e.g. Cohen's *d*, Pearson's *r*), indicating how they were calculated |
| | x | Clearly defined error bars<br>*State explicitly what error bars represent (e.g. SD, SE, CI)* |

*Our web collection on statistics for biologists may be useful.*

## Software and code

Policy information about availability of computer code

| | |
|---|---|
| Data collection | Attune Nxt Software 2 |
| Data analysis | FlowJo 9 (TreeStar); TASBE FlowAnalytics 6, Microsoft Excel 2011, MATLAB_R2017b |

For manuscripts utilizing custom algorithms or software that are central to the research but not yet described in published literature, software must be made available to editors/reviewers upon request. We strongly encourage code deposition in a community repository (e.g. GitHub). See the Nature Research guidelines for submitting code & software for further information.

## Data

Policy information about availability of data

All manuscripts must include a data availability statement. This statement should provide the following information, where applicable:
- Accession codes, unique identifiers, or web links for publicly available datasets
- A list of figures that have associated raw data
- A description of any restrictions on data availability

Data are available from the corresponding authors upon reasonable request. Plasmids and construction resources are available at https://www.addgene.org/browse/article/xxxxx/.

# Field-specific reporting

Please select the best fit for your research. If you are not sure, read the appropriate sections before making your selection.

- [x] Life sciences
- [ ] Behavioural & social sciences
- [ ] Ecological, evolutionary & environmental sciences

For a reference copy of the document with all sections, see nature.com/authors/policies/ReportingSummary-flat.pdf

# Life sciences study design

All studies must disclose on these points even when the disclosure is negative.

| | |
|---|---|
| Sample size | We chose to do three replicates for each experiments based on similar type genetic tool experiments in the literature. |
| Data exclusions | No data was excluded. |
| Replication | We replicated most of the experiments at least two times, achieving similar results. |
| Randomization | No randomization was used. All samples used were from the same homogeneous mix of cells and therefore eliminated possible covariates between different experimental groups. |
| Blinding | No blinding was used. Experiments used cell lines that should not be affected by the experimenter. |

# Reporting for specific materials, systems and methods

## Materials & experimental systems

| n/a | Involved in the study | |
|---|---|---|
| ☐ | ☒ | Unique biological materials |
| ☐ | ☐ | Antibodies |
| ☐ | ☒ | Eukaryotic cell lines |
| ☐ | ☐ | Palaeontology |
| ☐ | ☒ | Animals and other organisms |
| ☐ | ☐ | Human research participants |

## Methods

| n/a | Involved in the study | |
|---|---|---|
| ☐ | ☐ | ChIP-seq |
| ☐ | ☒ | Flow cytometry |
| ☐ | ☐ | MRI-based neuroimaging |

## Unique biological materials

Policy information about availability of materials

| | |
|---|---|
| Obtaining unique materials | Select DNA plasmids will available on repository Addgene |

## Antibodies

| | |
|---|---|
| Antibodies used | *Describe all antibodies used in the study; as applicable, provide supplier name, catalog number, clone name, and lot number.* |
| Validation | *Describe the validation of each primary antibody for the species and application, noting any validation statements on the manufacturer's website, relevant citations, antibody profiles in online databases, or data provided in the manuscript.* |

## Eukaryotic cell lines

Policy information about cell lines

| | |
|---|---|
| Cell line source(s) | ThermoFisher |
| Authentication | No authentication was made. Cells were purchased directly from source. |
| Mycoplasma contamination | We did not test for mycoplasma contamination. |

Commonly misidentified lines
(See ICLAC register)

HEK-derivative HEK293FT: An easy cell line to maintain and affords high transfection efficiencies.

## Palaeontology

Specimen provenance

*Provide provenance information for specimens and describe permits that were obtained for the work (including the name of the issuing authority, the date of issue, and any identifying information).*

Specimen deposition

*Indicate where the specimens have been deposited to permit free access by other researchers.*

Dating methods

*If new dates are provided, describe how they were obtained (e.g. collection, storage, sample pretreatment and measurement), where they were obtained (i.e. lab name), the calibration program and the protocol for quality assurance OR state that no new dates are provided.*

☐ Tick this box to confirm that the raw and calibrated dates are available in the paper or in Supplementary Information.

## Animals and other organisms

Policy information about studies involving animals; ARRIVE guidelines recommended for reporting animal research

Laboratory animals

Female NSG mice, 4-6 weeks of age, were purchased from Jackson Laboratories

Wild animals

*Provide details on animals observed in or captured in the field; report species, sex and age where possible. Describe how animals were caught and transported and what happened to captive animals after the study (if killed, explain why and describe method; if released, say where and when) OR state that the study did not involve wild animals.*

Field-collected samples

*For laboratory work with field-collected samples, describe all relevant parameters such as housing, maintenance, temperature, photoperiod and end-of-experiment protocol OR state that the study did not involve samples collected from the field.*

## Human research participants

Policy information about studies involving human research participants

Population characteristics

*Describe the covariate-relevant population characteristics of the human research participants (e.g. age, gender, genotypic information, past and current diagnosis and treatment categories). If you filled out the behavioural & social sciences study design questions and have nothing to add here, write "See above."*

Recruitment

*Describe how participants were recruited. Outline any potential self-selection bias or other biases that may be present and how these are likely to impact results.*

## ChIP-seq

### Data deposition

☐ Confirm that both raw and final processed data have been deposited in a public database such as GEO.

☐ Confirm that you have deposited or provided access to graph files (e.g. BED files) for the called peaks.

Data access links
*May remain private before publication.*

*For "Initial submission" or "Revised version" documents, provide reviewer access links. For your "Final submission" document, provide a link to the deposited data.*

Files in database submission

*Provide a list of all files available in the database submission.*

Genome browser session
(e.g. UCSC)

*Provide a link to an anonymized genome browser session for "Initial submission" and "Revised version" documents only, to enable peer review. Write "no longer applicable" for "Final submission" documents.*

### Methodology

Replicates

*Describe the experimental replicates, specifying number, type and replicate agreement.*

Sequencing depth

*Describe the sequencing depth for each experiment, providing the total number of reads, uniquely mapped reads, length of reads and whether they were paired- or single-end.*

Antibodies

*Describe the antibodies used for the ChIP-seq experiments; as applicable, provide supplier name, catalog number, clone name, and lot number.*

Peak calling parameters

*Specify the command line program and parameters used for read mapping and peak calling, including the ChIP, control and index files used.*

Data quality

*Describe the methods used to ensure data quality in full detail, including how many peaks are at FDR 5% and above 5-fold enrichment.*

Software | *Describe the software used to collect and analyze the ChIP-seq data. For custom code that has been deposited into a community repository, provide accession details.*

# Flow Cytometry

## Plots

Confirm that:

- [x] The axis labels state the marker and fluorochrome used (e.g. CD4-FITC).
- [x] The axis scales are clearly visible. Include numbers along axes only for bottom left plot of group (a 'group' is an analysis of identical markers).
- [x] All plots are contour plots with outliers or pseudocolor plots.
- [x] A numerical value for number of cells or percentage (with statistics) is provided.

## Methodology

Sample preparation: For all experiments, except for given times during time-course experiments, plates were removed from the incubator two days post-transfection. Cells were trypsinized using 0.05% trypsin/0.53 mM EDTA (Corning) and resuspended with cell culture medium to a volume of 200 μL in 96 U-bottom plates.

Instrument: All cell populations were analyzed using a ThermoFisher Attune Nxt cytometer equipped with four lasers. GFP was detected using a 488 nm blue laser and a 510/10 bandpass emission filter; a 530/30 bandpass filter was used for experiments that converted to MEFL units. mTagBFP was detected using a 405 nm violet laser and 440/50 bandpass emission filter. iRFP720 was detected using a 638 nm red laser and 720/30 bandpass emission filter.

Software: Experiments in Figure 2 were analyzed using TASBE Flow Analytics, an open source flow cytometry software tool. All other cytometry data was processed with FlowJo 9

Cell population abundance: We did not sort the cells.

Gating strategy: For FlowAnalytics: A two-component multi-dimensional gaussian mixture model is fit to FSC/SSC the negative control sample, segmenting the population into likely single-cell events vs. debris/cluster events. An mTagBFP fluorescent protein was used as a transfection marker and positive BFP cells (>10^6 molecules ) were first gated, then mean GFP fluorescence determined afterwards.
For FlowJo: A polygonal FSC-A/SSC-A gate was used to remove debris from the mammalian cell populations. An mTagBFP fluorescent protein was used as a transfection marker and positive BFP cells (based of a comparison to wildtype cells) were first gated, then mean GFP fluorescence determined afterwards.

- [x] Tick this box to confirm that a figure exemplifying the gating strategy is provided in the Supplementary Information.

# Magnetic resonance imaging

## Experimental design

Design type: *Indicate task or resting state; event-related or block design.*

Design specifications: *Specify the number of blocks, trials or experimental units per session and/or subject, and specify the length of each trial or block (if trials are blocked) and interval between trials.*

Behavioral performance measures: *State number and/or type of variables recorded (e.g. correct button press, response time) and what statistics were used to establish that the subjects were performing the task as expected (e.g. mean, range, and/or standard deviation across subjects).*

## Acquisition

Imaging type(s): *Specify: functional, structural, diffusion, perfusion.*

Field strength: *Specify in Tesla*

Sequence & imaging parameters: *Specify the pulse sequence type (gradient echo, spin echo, etc.), imaging type (EPI, spiral, etc.), field of view, matrix size, slice thickness, orientation and TE/TR/flip angle.*

Area of acquisition: *State whether a whole brain scan was used OR define the area of acquisition, describing how the region was determined.*

Diffusion MRI: [ ] Used [ ] Not used

## Preprocessing

Preprocessing software: *Provide detail on software version and revision number and on specific parameters (model/functions, brain extraction, segmentation, smoothing kernel size, etc.).*

Normalization

*If data were normalized/standardized, describe the approach(es): specify linear or non-linear and define image types used for transformation OR indicate that data were not normalized and explain rationale for lack of normalization.*

Normalization template

*Describe the template used for normalization/transformation, specifying subject space or group standardized space (e.g. original Talairach, MNI305, ICBM152) OR indicate that the data were not normalized.*

Noise and artifact removal

*Describe your procedure(s) for artifact and structured noise removal, specifying motion parameters, tissue signals and physiological signals (heart rate, respiration).*

Volume censoring

*Define your software and/or method and criteria for volume censoring, and state the extent of such censoring.*

## Statistical modeling & inference

Model type and settings

*Specify type (mass univariate, multivariate, RSA, predictive, etc.) and describe essential details of the model at the first and second levels (e.g. fixed, random or mixed effects; drift or auto-correlation).*

Effect(s) tested

*Define precise effect in terms of the task or stimulus conditions instead of psychological concepts and indicate whether ANOVA or factorial designs were used.*

Specify type of analysis: ☐ Whole brain ☐ ROI-based ☐ Both

Statistic type for inference
(See Eklund et al. 2016)

*Specify voxel-wise or cluster-wise and report all relevant parameters for cluster-wise methods.*

Correction

*Describe the type of correction and how it is obtained for multiple comparisons (e.g. FWE, FDR, permutation or Monte Carlo).*

## Models & analysis

| n/a | Involved in the study |
|---|---|
| ☐ | ☐ Functional and/or effective connectivity |
| ☐ | ☐ Graph analysis |
| ☐ | ☐ Multivariate modeling or predictive analysis |

Functional and/or effective connectivity

*Report the measures of dependence used and the model details (e.g. Pearson correlation, partial correlation, mutual information).*

Graph analysis

*Report the dependent variable and connectivity measure, specifying weighted graph or binarized graph, subject- or group-level, and the global and/or node summaries used (e.g. clustering coefficient, efficiency, etc.).*

Multivariate modeling and predictive analysis

*Specify independent variables, features extraction and dimension reduction, model, training and evaluation metrics.*
